# Atypically presented dysphagia in a patient with primary hyperparathyroidism that resolved after parathyroidectomy: A case report

**DOI:** 10.1016/j.ijscr.2024.110480

**Published:** 2024-10-18

**Authors:** Wasef Alhroub, Majd Oweidat, Mohammed Alra'e, Rami Yousef Alayasa

**Affiliations:** a Department of Internal Medicine, College of Medicine, Hebron University, Hebron, Palestine; b Department of Surgery, College of Medicine, Hebron University, Hebron, Palestine

**Keywords:** Hypercalcemia, Parathyroidectomy, Dysphagia resolution, Endocrine disorders, Tc-99 m sestamibi scan, Bone mineral density

## Abstract

**Introduction:**

Dysphagia is a rare initial manifestation of primary hyperparathyroidism, which typically presents with gastrointestinal symptoms like anorexia and constipation. This case presentation aims to describe swallowing difficulty as a potential primary symptom of parathyroid hormone (PTH)-mediated hypercalcemia.

**Presentation of case:**

An elderly Palestinian female presented with a two-year history of progressive dysphagia, along with mood disturbances and increased urination. Her medical history included osteoporosis and hyperparathyroidism. Laboratory tests showed elevated PTH and calcium levels. Imaging, including neck ultrasound (US) and Tc99m sestamibi scan, identified a parathyroid adenoma. Despite normal findings on esophagogastroduodenoscopy, a focused single parathyroidectomy was performed. Post-surgery, the patient's dysphagia resolved completely, and her calcium and PTH levels normalized.

**Discussion:**

This case highlights an atypical presentation of PTH-mediated hypercalcemia where dysphagia was the primary symptom. Literature review shows similar cases where parathyroid adenomas presented with unusual symptoms, emphasizing the need for thorough diagnostic evaluation. The resolution of dysphagia following surgery suggests a likely correlation between hypercalcemia and dysphagia, implying a possible causative association.

**Conclusion:**

This case demonstrates dysphagia as a rare but significant symptom of hypercalcemia in a patient with a small parathyroid adenoma. Considering its size, it was unlikely to cause dysphagia through mechanical obstruction. Thus, a functional cause linked to hypercalcemia was likely to be the cause of dysphagia. Surgical removal of the adenoma resulted in resolution of symptoms. A literature review was performed, which revealed similar cases of dysphagia related to hypercalcemia, supporting the fact that in this patient, dysphagia was related to the hypercalcemia.

## Highlights

• Dysphagia can be a rare, atypical presentation of primary hyperparathyroidism.
• Parathyroidectomy successfully resolved the patient's dysphagia symptoms.
• Diagnostic challenges arise when hyperparathyroidism presents with uncommon symptoms.
• Literature review highlights limited cases of dysphagia linked to parathyroid adenoma.

## 1 Introduction

Calcium, the predominant cation in the human organism, is essential for a multitude of physiological functions, including neurotransmission, enzyme function, and diverse cellular processes. Hypercalcemia can be broadly categorized into two major categories: parathyroid hormone-mediated and non-parathyroid hormone-mediated causes [1].

Primary hyperparathyroidism, predominantly caused by parathyroid adenomas, is a common endocrine disorder characterized by excessive secretion of parathyroid hormone (PTH), leading to hypercalcemia [2]. The condition's prevalence increases with age, particularly among postmenopausal women, and can manifest with a wide range of symptoms due to the systemic effects of elevated calcium levels [3]. While classic symptoms include fatigue, bone pain, renal calculi, and neuropsychiatric disturbances, gastrointestinal manifestations are also common [4].

However, dysphagia, particularly oropharyngeal dysphagia, is a rare and atypical presentation of hyperparathyroidism. This condition is typically associated with neurological or structural abnormalities of the oropharynx and esophagus. The association of dysphagia with primary hyperparathyroidism poses a diagnostic challenge, as it is an uncommon initial symptom and may lead to a delay in diagnosis [5].

This case report aims to emphasize the importance of considering PTH-mediated hypercalcemia in the differential diagnosis of dysphagia, especially in patients presenting with hypercalcemia and without obvious causes of esophageal dysfunction. The resolution of the functional dysphagia following focused single parathyroidectomy underscores the significance of early recognition and surgical intervention in managing hypercalcemia and its related complications.

Our work has been reported in line with the SCARE Guidelines 2023 criteria [6].

## 2 Presentation of case

A Palestinian female in her late 50s presented to our department with a decade-long history of difficulty swallowing solid foods, a symptom that had progressively worsened over the last two years. Her dysphagia was accompanied by mood disturbances and increased urination, suggestive of nephrogenic diabetes insipidus. The patient's medical history was significant for osteoporosis, managed subclinical hypothyroidism, and a five-year history of hyperparathyroidism with associated hypercalcemia. There was no family history of hypercalcemia, kidney stones, or fragility fractures.

On physical examination, her vital signs were stable, and the rest of the examination was unremarkable. Laboratory investigations revealed elevated parathyroid hormone (PTH) levels at 99.6 pg/ml and hypercalcemia at 11.5 mg/dl, with serum phosphorus at 2.25 mg/dl. Thyroid function tests were within normal limits.

**Fig. 1:**
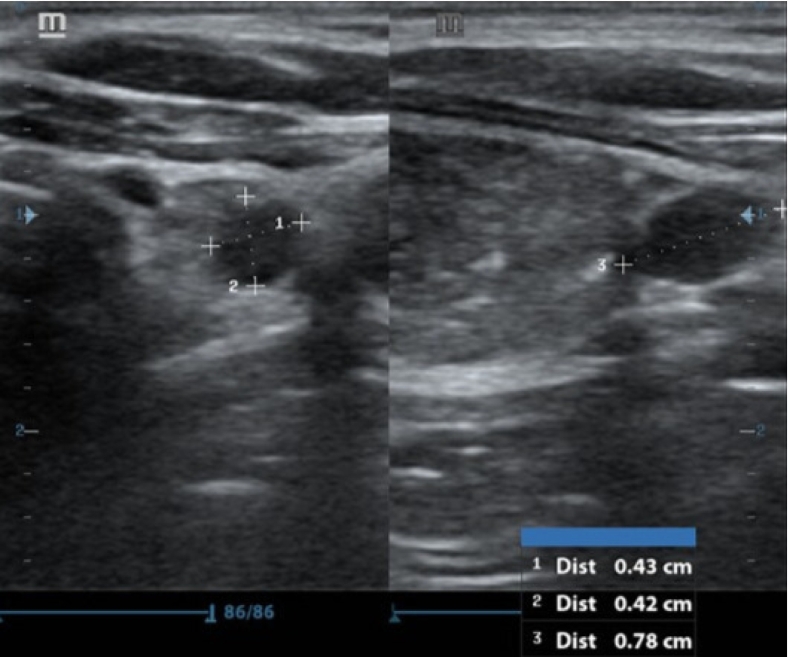
Ultrasound-guided exploration of the neck, showing a well-defined, homogenous, hypoechoic, oval-shaped solitary nodule below the lower pole of the left thyroid lobe, visualized from different angles.

**Fig. 2:**
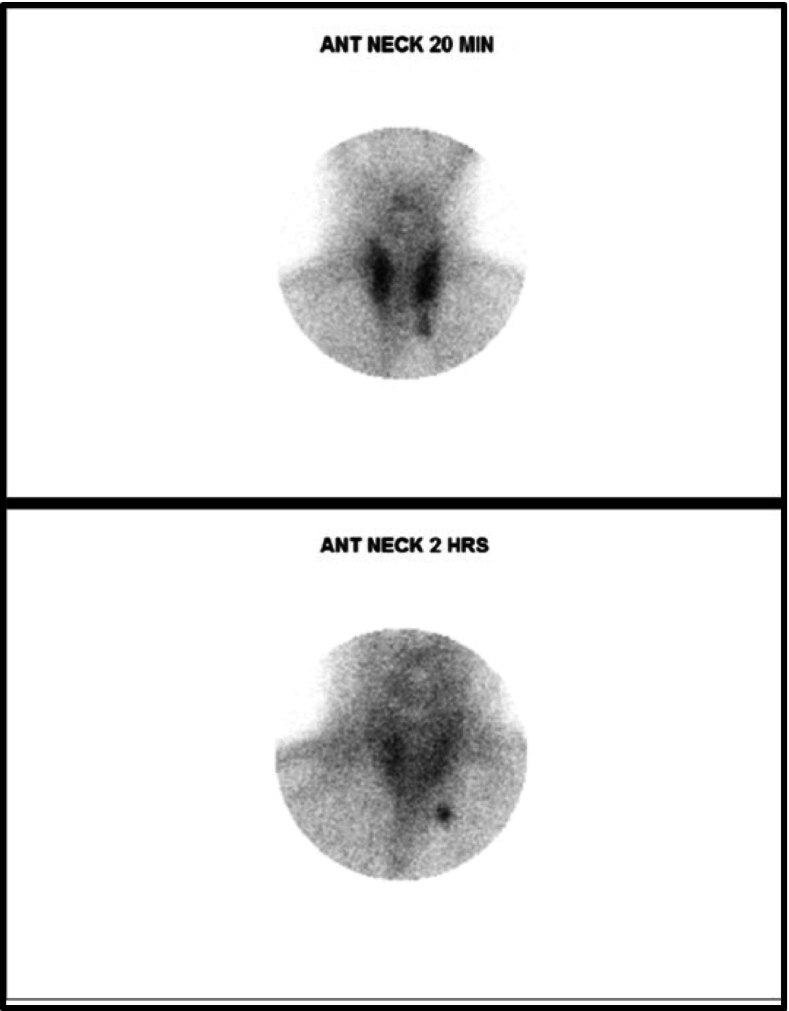
A nuclear medicine parathyroid scan (technetium-99 m sestamibi). Early (20 min) and delayed (2 h) images were taken following IV injection of Tc-99 m radioisotope. The early image revealed homogenous tracer uptake within the thyroid gland, with distinct focal prominent activity inferior to the lower pole of the left thyroid lobe. The delayed image revealed a persistent focus of tracer retention in the same area, while the remainder of the thyroid gland exhibited significant washout.

**Fig. 3:**
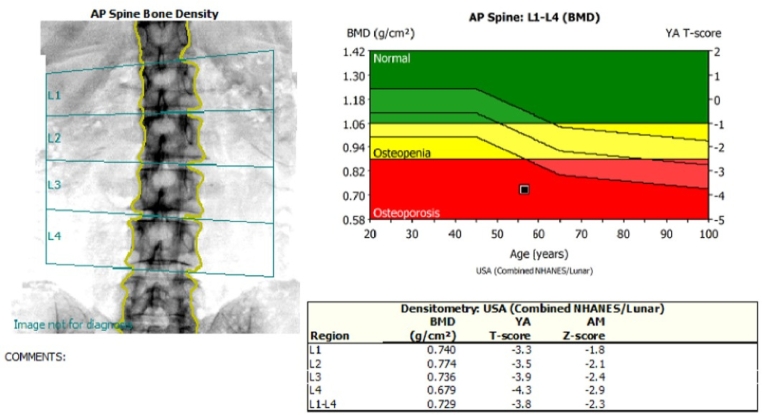
Bone mineral density (BMD) scan. This anteroposterior (AP) view of the lumbar spine (L1-L4) shows a measured BMD of 0.729 g/cm^2^, with a T-score of −3.8, consistent with osteoporosis according to WHO criteria.

A neck US identified a hypoechoic nodule below the lower pole of the left thyroid lobe measuring 0.43 * 0.42 * 0.78 cm^3^ (Fig. 1), with normal Doppler studies and no evidence of retrosternal extension or tracheal compression. Fine needle aspiration of the nodule revealed follicular cells in a colloid background. A Tc99m sestamibi scan showed homogenous uptake within the thyroid gland, with a distinct focal activity below the lower pole of the left thyroid lobe. The findings were compatible with tracer avid left inferior parathyroid lesion, suggesting an adenoma (Fig. 2). BMD of the spine revealed that the patient was osteoporotic according to World Health Organization (WHO) criteria (Fig. 3). Subsequent Single Photon Emission Computed Tomography (SPECT) confirmed the presence of a soft tissue lesion, measuring 6.6 × 3.8 mm, consistent with a left inferior parathyroid adenoma.

To further investigate the patient's dysphagia, an esophagogastroduodenoscopy was performed. The examination revealed a normal esophagus with no retained food or liquid, a normal gastroesophageal junction, and no signs of stricture, esophagitis, or hiatal hernia. Although multiple linear erosions and hemorrhagic spots were noted in the antrum of the stomach, the duodenum appeared unremarkable. However, this investigation did not provide a clear explanation for the dysphagia.

The patient underwent a focused single parathyroidectomy. Intraoperatively, a parathyroid adenoma was identified and successfully excised. Postoperative laboratory results showed normalization of serum calcium (9 mg/dl) and PTH levels (44.9 pg/ml). Post-operative barium swallow was normal. The diagnosis of parathyroid adenoma was confirmed via histopathological workup. The patient's symptoms, including dysphagia and those related to hypercalcemia, resolved completely within 24 h. Long-term follow-up over an 8-month period showed normal serum calcium levels with no signs of abnormalities. The patient's anonymity was preserved throughout the process, and informed consent was obtained for publication.

## 3 Discussion

This case highlights an atypical presentation of PTH-mediated hypercalcemia. The patient exhibited symptoms that deviated from the classic manifestations of hypercalcemia, prompting a detailed diagnostic approach. This case is particularly notable due to its distinct clinical features and the insights it provides into managing similar cases.

Parathyroid adenoma leads to excessive secretion of parathyroid hormone (PTH), disrupting calcium and phosphate balance [7]. Normally, PTH increases calcium absorption in the kidneys, stimulates bone resorption, and enhances intestinal calcium absorption via vitamin D activation. Overproduction of PTH results in elevated calcium levels and decreased phosphate, which underlies the symptoms seen in parathyroid disease [8].

Current guidelines recommend a targeted approach for diagnosing and managing parathyroid adenoma. Diagnosis typically begins with identifying hypercalcemia on routine blood tests, followed by PTH level assessment. Imaging techniques, particularly sestamibi SPECT combined with US are used for accurate localization [[9], [10], [11]]. Parathyroidectomy is the only definitive treatment for primary hyperparathyroidism, with the majority of patients having a single parathyroid adenoma [12]. Patients with a well-localized single adenoma like our patient have a focused parathyroidectomy, which has a smaller incision and requires less dissection. Focused parathyroidectomy offers much lower overall complication rates and a shorter operating duration than bilateral parathyroid exploration [13]. In focused parathyroidectomy, intraoperative localization of the abnormal gland can be achieved using US or a gamma probe [12].

Differential diagnoses for our patient's presentation included cricopharyngeal achalasia, spastic motor disorders such as diffuse esophageal spasm, hypertensive lower esophageal sphincter, and nutcracker esophagus, and obstructive lesions, all of which were ruled out by barium swallow, esophagogastroduodenoscopy, CT scan, and neck US.

Other possibilities included a cerebrovascular accident, brainstem tumors, degenerative diseases like amyotrophic lateral sclerosis and multiple sclerosis, all of which were ruled out with a CT scan, history, and physical examination. Scleroderma was also ruled out because there were no other pertinent manifestations. Myasthenia gravis and dermatomyositis were ruled out by history and physical examination.

In reviewing the literature on parathyroid adenomas and their presentations, several noteworthy cases highlight the diverse manifestations of this condition. One report described a 62-year-old man with severe dysphagia and significant weight loss, revealing a large parathyroid adenoma causing esophageal compression and contributing to swallowing difficulties [5]. Similarly, another case reported a 72-year-old patient with extrapyramidal symptoms and dysphagia secondary to multiple atypical parathyroid tumors, which progressed to a hypercalcemic crisis requiring urgent intervention [14]. In a different instance, a 68-year-old female presented with anorexia, mutism, and dysphagia, eventually diagnosed with a retropharyngeal ectopic parathyroid adenoma, with symptoms resolving post-treatment [15]. There was also a rare instance of a non-functional cystic parathyroid adenoma in a 52-year-old female, emphasizing the need for surgical intervention despite its non-secreting nature [16]. Another case discussed a patient with dysphagia and odynophagia but normal calcium and PTH levels, where an infarcted parathyroid adenoma was eventually identified and surgically treated [17]. A spontaneous cervical hemorrhage from an extra-capsular parathyroid adenoma presented with sore throat and dysphagia, underscoring the diagnostic value of serum calcium in acute presentations [18]. Insights were also provided from a case of primary hyperparathyroidism with dysphagia, detailing the multifaceted nature of the disease [19]. Another report described a 51-year-old female with persistent hyperparathyroidism and dysphagia, found to have parathyroid carcinoma, emphasizing the importance of distinguishing between adenoma and carcinoma through histological examination [20]. In another case, a 69-year-old woman with anterior cervical hematoma and dysphagia was ultimately diagnosed with a parathyroid adenoma [21]. Finally, there was a unique instance of bilateral parathyroid adenomas located in the pyriform sinuses, presenting with dysphagia and successfully treated with surgical excision [22]. These cases collectively demonstrate the wide range of symptoms associated with parathyroid adenomas, from dysphagia and neurological symptoms to acute hemorrhagic presentations, illustrating the critical importance of comprehensive diagnostic evaluation and treatment.

In this case, the clinical symptoms of hypercalcemia correlated well with the pathological findings of a parathyroid adenoma, with imaging results accurately guiding the surgical intervention and confirming the presence of the adenoma. Resolution of functional dysphagia occurs concurrently with resolution of hypercalcemia after surgical treatment of primary hyperparathyroidism. The pathophysiology of dysphagia in hypercalcemia remains incompletely understood [5].We believe that our patient's functional dysphagia was caused by hypercalcemia, which resolved following parathyroidectomy. This suggests a probable correlation between hypercalcemia and dysphagia, indicating a potential causal relationship.

Calcium is crucial for nerve conduction, transmission, and muscle contraction [23]. The pathophysiologic mechanism of hypercalcemia-induced severe dysphagia can be attributed to calcium ion imbalance affecting neuromuscular transmission and esophageal muscle contraction. A rapid influx of calcium ions at the neuromuscular synapse triggers excessive acetylcholine release, which in turn disrupts normal muscle function, leading to paralysis of the esophageal muscles [5].

Postoperative outcomes were consistent with expectations based on current clinical guidelines, underscoring the effective correlation between clinical symptoms and pathological findings. These findings reinforce the importance of timely diagnosis and intervention in parathyroid adenoma, though it remains crucial to be vigilant for potential complications and ensure thorough postoperative monitoring. This case highlights the need for continued adherence to established guidelines to optimize patient outcomes.

## 4 Conclusion

Although dysphagia is an uncommon presentation of parathyroid adenoma, this case illustrates its potential significance in patients with hypercalcemia. Our case describes a patient diagnosed with a solitary, relatively small parathyroid adenoma. Dysphagia in this patient is unlikely to be caused by mechanical mass effect of the adenoma, due to its small size and negative investigations for obstructing lesions, making the diagnosis of a functional cause more likely. Managing the patient with recommended guidelines, by resection of the adenoma resulted in resolution of dysphagia. This outcome suggests a potential connection between hypercalcemia and dysphagia, indicating a possible causal relationship. We find that hypercalcemia is associated with dysphagia in similar case reports, making the functional dysphagia in this patient likely to be caused by hypercalcemia.

## Registration of research studies

N/A.

## Guarantor

Majd Oweidat

## CRediT authorship contribution statement

W.A. handled conceptualization, data curation, and software. M.O. contributed to writing, editing and reviewing of the original draft. M.A. contributed to the investigation, visualization, management of resources and validation. R.A. provided supervision.

## Consent

Written informed consent was obtained from the patient for publication of this case report and accompanying images. A copy of the written consent is available for review by the Editor-in-Chief of this journal on request.

## Ethical approval

Our institution has granted ethical approval for this study.

## Sources of funding

There are no funding sources.

## Declaration of competing interest

None.
